# Nationwide Study of Drug Resistance Mutations in HIV-1 Infected Individuals under Antiretroviral Therapy in Brazil

**DOI:** 10.3390/ijms22105304

**Published:** 2021-05-18

**Authors:** Ana Santos-Pereira, Vera Triunfante, Pedro M. M. Araújo, Joana Martins, Helena Soares, Eva Poveda, Bernardino Souto, Nuno S. Osório

**Affiliations:** 1 Life and Health Sciences Research Institute (ICVS), School of Medicine, University of Minho, 4710-057 Braga, Portugal; anaapspereira@gmail.com (A.S.-P.); vera.triunfante@gmail.com (V.T.); id5827@alunos.uminho.pt (P.M.M.A.); joana_smar@hotmail.com (J.M.); bernardino@ufscar.br (B.S.); 2 ICVS/3B’s-PT Government Associate Laboratory, 4710-057 Braga, Portugal; 3 Human Immunobiology and Pathogenesis Laboratory, 1150-082 Lisbon, Portugal; helena.soares@nms.unl.pt; 4 CEDOC-Chronic Diseases Research Center, NOVA Medical School, Faculdade de Ciências Médicas, NOVA University of Lisbon, 1150-082 Lisbon, Portugal; 5 Group of Virology and Pathogenesis, Galicia Sur Health Research Institute (IIS Galicia Sur)-Complexo Hospitalario Universitario de Vigo, SERGAS-UVigo, 36213 Vigo, Spain; Eva.Poveda.Lopez@sergas.es; 6 Department of Medicine, Federal University of São Carlos, São Paulo 13565-905, Brazil

**Keywords:** HIV-1, drug resistance mutations, K65R, antiretroviral treatment failure, tenofovir, Brazilian cohort study

## Abstract

The success of antiretroviral treatment (ART) is threatened by the emergence of drug resistance mutations (DRM). Since Brazil presents the largest number of people living with HIV (PLWH) in South America we aimed at understanding the dynamics of DRM in this country. We analyzed a total of 20,226 HIV-1 sequences collected from PLWH undergoing ART between 2008–2017. Results show a mild decline of DRM over the years but an increase of the K65R reverse transcriptase mutation from 2.23% to 12.11%. This increase gradually occurred following alterations in the ART regimens replacing zidovudine (AZT) with tenofovir (TDF). PLWH harboring the K65R had significantly higher viral loads than those without this mutation (*p* < 0.001). Among the two most prevalent HIV-1 subtypes (B and C) there was a significant (*p* < 0.001) association of K65R with subtype C (11.26%) when compared with subtype B (9.27%). Nonetheless, evidence for K65R transmission in Brazil was found both for C and B subtypes. Additionally, artificial neural network-based immunoinformatic predictions suggest that K65R could enhance viral recognition by HLA-B27 that has relatively low prevalence in the Brazilian population. Overall, the results suggest that tenofovir-based regimens need to be carefully monitored particularly in settings with subtype C and specific HLA profiles.

## 1. Introduction

According to the Joint United Nations Programme on HIV/AIDS (UNAIDS), 690,000 individuals died in 2019 of human immunodeficiency virus (HIV) infection-related causes. Although the global number of new infections (1.7 million) has been reduced by 23%, since 2010, Latin America has presented a 21% increase. Particularly, Brazil experienced an increase of 17%, with 48,000 new reported infections and 14,000 acquired immunodeficiency syndrome (AIDS)-related deaths, in the same year [1].

Antiretroviral therapy (ART) significantly improves patients’ survival, increasing life expectancy and quality [2,3,4]. Furthermore, it plays a relevant role in the prevention of HIV-1 transmission in the population [5,6,7,8]. However, the emergence of drug resistance mutations (DRM) represents a major threat for the continued control of HIV replication, and consequent potential increase in the transmission of viral strains with DRM [9,10]. The prevalence of DRM can greatly vary between different geographical areas, depending on several factors, ranging from study design to the sociodemographic characteristics of the population and most prevalent HIV-1 subtypes [9,11,12,13,14,15]. With a total area as vast as 8.51 million km^2^, different DRM rates have also been found in distinct Brazilian geographic regions [9,12,16,17,18,19,20].

In 1996, Brazil became the first middle-income country to ensure free access to ART to all individuals infected with HIV, through the public Unified Health System [21,22]. Currently, 69% of the people living with HIV are under ART [1]. Initial ART regimens in Brazil consist of a combination of three drugs, comprising two NRTIs and a third of different ART class: a non-nucleoside reverse transcriptase inhibitor (NNRTI), a protease inhibitor (PI) or an integrase inhibitor (INSTI) [21]. Although pre-treatment testing for DRM in the reverse transcriptase (RT) and protease (PR) has been widely recommended by the International Antiviral Society–USA, the US Department of Health and Human Services and the European AIDS Clinical Society, among others, viral genotyping and resistance testing are preferably performed in Brazil after 6 months of treatment failure [21,23,24,25]. After 2013, pre-treatment genotyping was also indicated by the Brazilian Government in the following scenarios: *Mycobacterium tuberculosis* (*Mtb*) co-infection, pregnant woman, children and new diagnoses with a sexual partner under ART [21,22]. Importantly, Brazil is the only country in South America that provides pre-exposure prophylaxis (PrEP) through the public health system [1,21].

Among the surveillance drug-resistance mutations (SDRM) [26], K65R is commonly associated with tenofovir (TDF) resistance and different prevalence rates of this mutation have been reported in several Brazilian cities [11,17,27]. It was also associated with resistance to other reverse transcriptase inhibitors (NRTIs), but it confers increased susceptibility to zidovudine (AZT) [28,29,30,31,32,33]. K65R is acquired in B and several other HIV-1 subtypes by the AAA→AGA substitution while in subtype C the involved substitution is AAG→AGG, which has been associated with higher probability of C subtype viruses to acquire this mutation [31,32,34,35].

The relevant reforms made in ART protocols in Brazil during the last decades, promoting the preferential use TDF containing schemes in the absence of universal HIV-1 genotyping, raised the hypothesis that the HIV DRM profile could have changed, bringing novel treatment obstacles and clinical implications. To test this hypothesis, we retrospectively studied data from 20,226 HIV-1 infected and ART-treated individuals from all regions of Brazil, from 2008 to 2017. We used statistical and genome sequence analysis to understand, at an unprecedented scale in Brazil, the dynamics of evolution of the HIV-1 drug resistance profiles. Additionally, we also relied on immunoinformatics to investigate factors possible underlying treatment failure and transmission of DRM.

## 2. Results

### 2.1. Characterization of the Study Population

The study comprised a total of 20,226 HIV-1 infected individuals, with a HIV-1 sequence genotyped between 2008 and 2017, on ART treatment, from which 44.3% (*n* = 8962) were female and 55.7% (*n* = 11,263) male. The main characteristics of the study population are shown in Table 1. The median age was 39.55 (±12.71) years (yrs). Most of the individuals were were between 30 and 49 yrs old (61.44%), from the State of São Paulo (21.98%; *n* = 4445), followed by the States of Rio Grande do Sul (11.96%; *n* = 2419), Minas Gerais (9.29%; *n* = 1878), Rio de Janeiro (9.20%; *n* = 1861) and Paraná (7.94%; *n* = 1606). The geographic frequency distribution for PLWH in the study population was in agreement with the official Brazilian HIV-1 prevalence reports [36]. These patients were on ART, in average, for 2.98 (±2.96) yrs and the most common treatment schemes combinations were: lamivudine (3TC)/efavirenz (EFV)/TDF (21.38%; *n* = 4324), 3TC/AZT/EFV (19.79%; *n* = 4003), 3TC/AZT/low-dose ritonavir-boosted lopinavir (LPV) (11.16%; *n* = 2258), 3TC/LPV/TDF (8.24%; *n* = 1666) and 3TC/atazanavir (ATV)/ritonavir (RTV)/TDF (6.70%; *n* = 1355).

### 2.2. Prevalence of Surveillance Drug-Resistance Mutations

**Figure 1:**
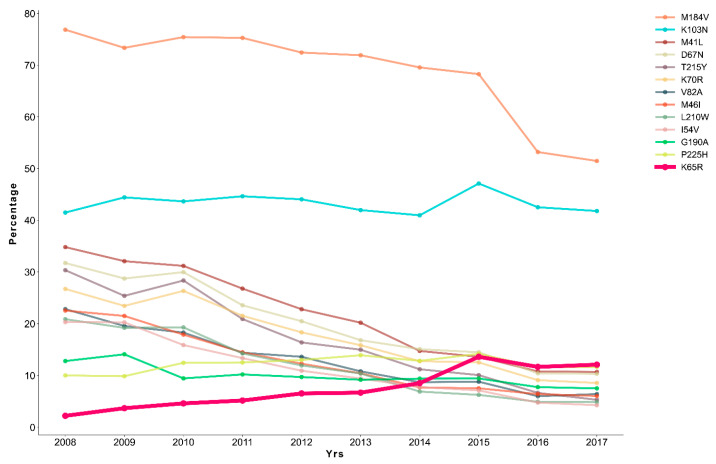
Surveillance drug-resistance mutations prevalence in HIV-1 RT and PR through the yrs 2008 to 2017. Percentage of individuals infected with a viral strain that presented one of the most common SDRM in the different yrs.

We evaluated the presence of surveillance drug-resistance mutations (SDRM) in the study population, including SDRM found isolated or in combinations in the same virus, and uncovered an overall prevalence of 84.10% (*n* = 17,011). SDRM were mostly found at the RT (83.24%; *n* = 16,836) and included 13,845 sequences with NRTI DRM and 11,720 sequences with NNRTI DRM. The most common SDRM (Figure 1, Supplementary Table S2) were the substitutions in RT amino acids M184V (65.53%, *n* = 13,265), K103N (40.20%, *n* = 8738), and M41L (17.21%, *n* = 3480). SDRM in PR were found in 5021 (24.82%) sequences and the more frequent were V82A (9.99%, *n* = 2021), M46I (9.58%, *n* = 1938), and I54V (8.50%, *n* = 1719).

### 2.3. A Gradual Alteration on the Prevalence of Surveillance Drug-Resistance Mutations

To investigate the temporal dynamics and evolution of the SDRM landscape in the study population we evaluated yearly SDRM prevalence from 2008 until 2017. The most common mutation across the years was M184V (Figure 1), notwithstanding a decreasing trend and a significant decrease between 2015 (68.29%, *n* = 1874) and 2016 (53.21%, *n* = 2684) (*p* < 0.001) (Table 2). Similarly, all the other 10 most frequent SDRM followed a decreasing trend along the years with the remarkable exception for K65R and K103N (Figure 1). K103N remained stable in the studied period. While the prevalence of viruses harboring K103N increased between 2014 (40.97%, *n* = 1180) and 2015 (47.12%, *n* = 1293; *p* < 0.001) this was accompanied with a significant decrease in 2016 (42.53%, *n* = 2145; *p* < 0.001) and no significant difference between 2008 and 2017 (*p* = 0.919). Importantly, K65R showed a clear rise along the years (Figure 1). In 2008, the mutation was relatively rare and only found in 2.23% (*n* = 8) of the population. Subsequently, this mutation became more prevalent and increased significantly between 2013 and 2015 (*p* < 0.001). Although its prevalence suffered a decrease between 2015 and 2016 (*p* = 0.0124), K65R prevailed the third most common SDRM in 2016 and 2017 (Figure 1). Overall, the data strongly supports that, in contrast with other prevalent SDRM, K65R prevalence was increasing in Brazil, raising additional concerns about the efficacy of some of the ART combinations used. In line with this hypothesis was the finding that viral loads for PLWH with K65R were significantly higher than for the rest of the cohort (180,739.60 ± 497,000.10 cop/mL vs. 95,358.26 ± 338,930.67 cop/mL, respectively; *p* < 0.001).

### 2.4. A Shift on Treatment Scheme during the Years

**Figure 2:**
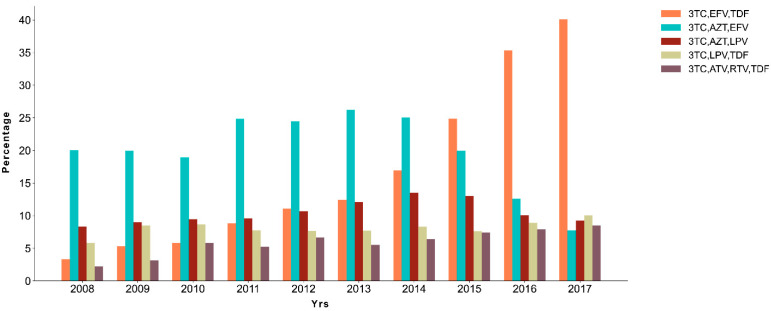
Treatment scheme used at time of viral sequencing between the yrs 2008 and 2017. Percentage of patients (*n* = 20,226) under the five more common ART combinations (3TC/EFV/TDF; 3TC/AZT/EFV; 3TC/AZT/LPV; 3TC/LPV/TDF; 3TC/ATV/RTV/TDF) in the year of the viral sequencing.

Having observed the clear increase in K65R along the studied yrs, we then decided to evaluate the ART combination schemes in use during the studied period (Figure 2), aiming at understanding ART usage impact on the SDRM. In 2008, from a total of 359 patients, the most prevalent ART combination was 3TC/AZT/EFV (20.06%, *n* = 72), followed by 3TC/AZT/LPV (8.36%, *n* = 30), prevailing the most common treatment till 2014 (25.03%, *n* = 721) among 2880 patients. From 2015 forward, 3TC/EFV/TDF scheme became the most prevalent. In this year (*n* = 2744), 24.85% (682) individuals were under 3TC/EFV/TDF drug combination and 19.97% (*n* = 548) under 3TC/AZT/EFV. In 2017, 3TC/EFV/TDF combination was used on 40.10% (*n* = 543) of the individuals on ART (*n* = 1354).

**Figure 3:**
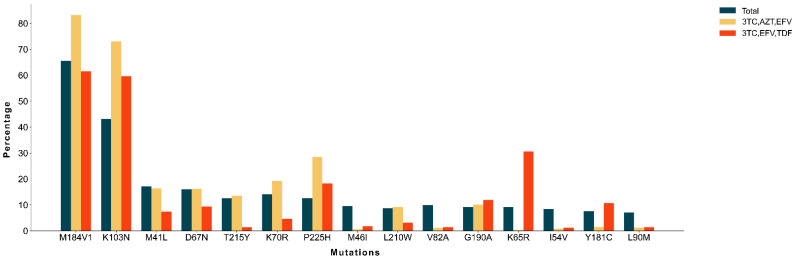
Most found SDRM by ART scheme. Percentage of individuals infected with viruses harboring common SDRM separated by ART scheme, discriminating 3TC/EFV/TDF (*n* = 4324, orange), 3TC/AZT/EFV (*n* = 4003, yellow) and in the total of ART schemes.

As we have found a shift on the ART treatment scheme during the studied years, we decided to evaluate SDRM prevalence separating the individuals using the most common drug combinations, 3TC/EFV/TDF (21.38%, *n* = 4324) and 3TC/AZT/EFV (19.79%, *n* = 4003) (Figure 3). The SDRM with most similar prevalence when comparing both groups were M46I, V82A, L90M and I54V. In contrast, K65R was only found in 0.37% (*n* = 15) of the viruses isolated from patients treated with 3TC/AZT/EFV vs. 30.64% (*n* = 1325) of the viruses from individuals using 3TC/EFV/TDF. This strong association of K65R with TDF containing schemes was found across HIV-1 subtypes (Supplementary Figure S1). Focusing on 2015, the year where the number of patients (*n* = 2744) using the two schemes was more even (3TC/AZT/EFV: 19.97%, *n* = 548; vs. 3TC/EFV/TDF: 24.85%, *n* = 682), 5 patients (0.91%) treated with 3TC/AZT/EFV were infected with a K65R mutant, being this number significantly different (*p* < 0.001) from the 267 patients (39.15%) treated with 3TC/EFV/TDF combination. Also, comparing all patients revealed a significant association between 3TC/EFV/TDF and K65R (*p* < 0.001).

### 2.5. Evidence for Transmission of K65R

**Figure 4:**
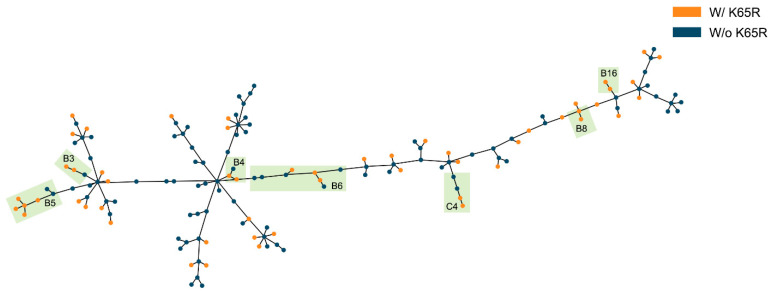
Minimum spanning network analysis of transmission clusters with more than one sequence with K65R mutation. Clusters compatible with K65R transmission are highlighted in green. Sequences without K65R mutation are marked in blue and in orange K65R mutants.

To investigate if the transmission of virus harboring K65R could have contributed to the increase in the prevalence of this mutation in Brazil, we performed a phylogenetic analysis on a subset of viral sequences including all the sequences with the K65R mutation and closely related sequences from public databases (Supplementary Figures S2 and S3). The definition of transmission clusters was performed as previously [37] considering tree branch statistical support and mean genetic distance criteria. We found K65R in at least two HIV-1 sequences in 21 well-delimited transmission clusters (16 from subtype B and 5 from subtype C) (Supplementary Table S1). The size of the inferred transmission clusters ranged from three to 16 individuals. The minimum spanning network analysis of these clusters (Figure 4) supports the occurrence of events of K65R transmission in the study cohort. Furthermore, the analysis of the clinical records from the individuals likely involved in K65R transmission supports this possibility for several cases showing a coincidence of geographic location, proximity in diagnostic dates and similar reported transmission routes (Supplementary Table S1). Despite the significant increased prevalence of the K65R in subtype C (11.26%, *n* = 347) viruses when compared with B (9.27%, *n*= 1305) subtype (*p* < 0.001, Supplementary Figure S2) we found probable events of K65R transmission also in subtype B viruses.

We then used an immunoinformatic approach to investigate the predicted impact of K65R on immune-driven selective pressures. We performed artificial neural network-based predictions of binding to the globally most frequent class I HLAs of all the peptide sequences overlapping K65R and ranging from eight to 12 amino acids with the mutant or the wild-type residue. Interestingly, we found that K65R overlapping peptides were predicted to be recognized by two HLAs (HLA-A03, HLA-B58) in the wildtype version and by one additional HLA (HLA-B27) in the mutant version (Table 3). HLA-B27 has the lowest prevalence (2.23%) in the Brazilian population of all the HLAs tested. Overall, our results suggest that the presence of K65R mutation might contribute for the immune control of the virus in individuals harboring HLA-B27 possibly decreasing its transmission in populations with high prevalence of this HLA.

## 3. Discussion

Acquired and transmitted DRM remain one of the major obstacles towards ART efficacy and AIDS treatment. In case of Brazil, previous regional studies showed distinct moderate patters of transmitted DRM prevalence [11,16,20,27,38], and high DRM rates in patients receiving ART [17,18,19,27,39]. In this study, we aimed at understanding SDRM [26] prevalence across the 27 Brazilian federative units focusing on 20,226 HIV-1 infected patients under different ART schemes, with viral sequence genotyped between 2008 and 2017. In accordance to the Brazilian HIV-1 epidemiology report [36], our cohort was composed mainly by male individuals (55.7%) and the majority of the patients’ age varied between 30 and 49 years old (61.44%).

A high prevalence of SDRM was observed with 84.1% of the infected individuals presenting at least one drug resistance mutation. Nonetheless, 68.5% of the genotyped sequences had a SDRM to NRTIs, a value that represents a decrease to what was previously reported in the literature [17,18,19,39,40], following a trend that has been described by Duani et al. and Diaz et al. in previous years [17,40]. However, NNRTI resistance rates (57.95%) did not follow the increasing trend reported by the same authors, presenting a similar value to what has been found in previous works [18,40]. PI resistance mutations (24.82%) also seem to follow the reported decreasing trend [17,40].

Also in line with previous studies is the finding of M184V (65.53%), K103N (40.20%), and M41L (17.21%) as the most common SDRM [17,19,39,40]. M184V, the most prevalent, is a NRTI mutation selected by 3TC and associated with impaired viral fitness and hypersensitization to other NRTI, including AZT and TDF [41,42,43]. Along with the most common mutations, M184V rates presented a decrease over the yrs (76.88% in 2008 to 51.48% in 2017). This decrease on acquired DRM was also observed in other countries and was hypothesized to be due to an improvement on treatment efficacy and also an increased accessibility to ART which enhances patient adherence to therapy [44,45,46]. Moreover, in Brazil, pretreatment genotyping has been extended to children living with HIV under 12 years of age, in case of *Mtb* co-infection, to pregnant woman and new diagnoses with a sexual partner on ART, since 2013 [47], which may had an impact the on the SDRM general decrease trend.

Nonetheless, it is important to highlight that the percentage of PLWH infected with a virus harboring M148V remained very high and above 50% after 2013. Moreover, K103N and P225H, both frequent NNRTI SDRM, remained stable over the yrs. K103N was previously reported as one of the most commonly acquired SDRM in Brazil [17,18,39,40] in association with the use of EFV [48]. P225H is also selected by EFV and generally occurs in the presence of K103N, synergically increasing EFV resistance [48,49,50]. As for K65R, it is selected by TDF and by other NRTI including abacavir (ABC) and 3TC, decreasing viral susceptibility to most of these drugs [29,31,51].

Previous work reported that K65R mutation presented varied prevalence distribution throughout time in different Brazilian regions [17,19,27]. Strikingly, in our nationwide work in Brazil we found that K65R was the only SDRM that followed an accentuated increase in prevalence over the studied years (2.23% in 2008 to 12.11% in 2017). The increasing and preferential usage of TDF in the clinical practice [21], including in a context of a failing regimen, could be the primordial reason for the significant expansion of K65R as other studies show a higher prevalence of this mutation in patients failing ART treatment [15,52,53,54,55]. However, although an increasing trend was identified in a cohort from India [56], several studies report a decrease on K65R levels over the years in other countries [48,57,58,59,60]. Reinheimer and colleagues suggest that the decline on K65R prevalence rates is linked with the increasing usage of single tablet ART regimens and the inclusion of AZT on the used treatment regimen, which has been liked with K65R development suppression [61]. Moreover, Theys et al. work [59] suggests that the found decline of tenofovir K65R selection rate in Portugal, between 2002 and 2010, is mainly caused by changes on treatment guidelines over the years and by the increased usage of combination of TDF and emtricitabine (FTC) [59]. Collectively, these studies suggest that the genetic or sociodemographic characteristics of the population treated with TDF might be influencing the K65R levels.

In 2019, 15.074 individuals were reported to use PrEP in Brazil [1]. Although PrEP consists of a co-formulation of FTC and TDF [62], evidence suggests that the risk of selection for TDF resistance is low and is more likely to occur in cases of undiagnosed HIV infection [63,64]. However, in these cases, high prevalence rates of M184V, which is also selected by FTC, and K65R as found in our study population, might compromise the efficacy of PrEP [65,66].

K65R has been associated with diminished viral fitness and replication, which has been linked with decreased transmission capacity [67,68]. Even though several studies support almost inexistent K65R transmission rates [11,69,70], Rhee and co-works present evidence of higher levels of transmitted K65R, especially in low- and middle-income countries [71]. In our phylogenetic analysis we found 21 transmission clusters containing at least two sequences with the K65R mutation. Moreover, minimum spanning network analysis supported the occurrence of events of K65R transmission in at least seven of these clusters. This might be suggestive that the efficacy of PrEP might be compromised in this setting. Indeed, we found much higher viral loads in patients harboring K65R mutation when compared with the rest of our cohort population. Similar viral loads were observed between individuals with and without tenofovir resistance by The TenoRes Study Group [72], which can be associated with increased mutation transmission. Furthermore, our results suggest that K65R overlapping peptides can be increasingly recognized by HLA-B27, a HLA that is linked with lower viral loads and slower diseased progression to AIDS [73,74]. The fact that Brazil presents a low prevalence of the HLA-B27, when in comparison with other regions [75,76,77], might have contributed to K65R expansion in this country. However, it is relevant to point-out that our results are based on in-silico prediction and not taking in consideration the possible impact on immune responses of the combination of different mutations in the same virus. Thus, it is relevant to address this topic in the future by performing functional studies.

Despite of the observed decreasing trend in the prevalence of some major SDRM in the studied Brazilian cohort, a high prevalence of these mutations was still verified. Indeed, our results show that the alteration performed in 2013 enlarging the criteria to include more PLWH in the recommendation for baseline SDRM testing had a positive impact but was still insufficient. We propose that the lack of universal baseline HIV-1 DRM screening to inform on effective ART regimens resulted in high levels of SDRM, such as M184V, K103N, and M41L underlying many cases of treatment failure in Brazil not only from 2008–2012 but also continuing from 2013–2017. Furthermore, we observed a clear increase in K65R reverse transcriptase SDRM that is an additional problem in Brazil that could have been aggravated by the circulation of HIV-1 subtype C and the HLA class I makeup of the population. This increase in K65R was mainly found in association with the use of TDF and is particularly relevant in combination with the high M184V levels found in the study population suggesting that the efficacy of PrEP might be compromised.

Overall, our results support that some of the drugs most frequently used in Brazil might be compromised due to the high frequency of SDRM and that baseline drug resistance testing should be universal and mandatory as it is the best way to promote personalized selection of the most optimized ART regiment.

## 4. Materials and Methods

### 4.1. Study Population

The collection of the patient’s data was performed anonymously after approval by the Brazilian national ethic committee through the protocol CAAE 53757016.0.0000.5504. All records from HIV-1-infected individuals followed by the Specialized Assistance Services on Sexually Transmissible Diseases and HIV/AIDS obtained at all 27 Brazilian federative units from 01/01/2008 to 04/30/2017 were curated in a relational database. A total of 20,226 HIV-1 infected patients were selected for this study according to the following inclusion criteria: (i) be under ART treatment; (ii) have at least one associated partial HIV-1 genome sequence available (complete HIV protease (*n* = 20,223) and complete reverse transcriptase (*n* = 20,214)). HIV-1 sequencing was performed as part of the routine clinical testing with commercially available HIV-1 genotyping systems based on Sanger sequencing.

### 4.2. HIV-1 Subtypes and Drug Resistance Mutations

HIV-1 subtypes were identified by relying on the consensus from least two of the three utilized subtyping tools: SNAPPY [78], jpHMM [79] and Stanford HIV Drug Resistance database (https://hivdb.stanford.edu/, accessed on 1 May 2021). All sequences were also analyzed using the Stanford HIVdb Genotypic Resistance Interpretation Algorithm to evaluate and interpret the presence of DRM in each sequence.

### 4.3. Phylogenetic Analysis of K65R Sequences and Transmission Clusters

All sequences having the K65R were separated by subtype and used to query local and public databases to identify highly related HIV-1 sequences. The resulting sequences from the most common subtypes B and C were aligned with MAFFT v7.309 [80] and used to make a phylogenetic reconstruction using PhyML v3.0 [37]. The best fitting substitution model was GTR + G4 + I, determined by PhyML SMS using AIC [81]. The heuristic trees search was performed using SPR methods. Bayesian evolutionary analyses were performed using BEAST v1.10.4 [82,83] with GTR + G4 + I, as the nucleotide substitution model. This phylogenetic representation was used to infer the transmission clusters as previously [84]. Minimum spanning network analysis of the sequences identified in transmission clusters with more than one K65R mutant was performed with PHYLOViZ [85].

### 4.4. HLA Binding Affinity Predictions

Predictions of binding for different class 1 human leukocyte antigens (HLA) were performed with NetMHCpan 4.1 [86]. The wildtype sequence (NPYNTPVFAIKKKDSTKWRKLVD) and the sequence with the K65R mutation (NPYNTPVFAIKRKDSTKWRKLVD) were used to generate all possible peptides sequences overlapping position 65 and ranging from eight to 12 amino acids. A group of 11 HLAs were used as supertype representatives: HLA-A01:01; HLA-A02:01; HLA-A03:01; HLA-A24:02; HLA-A26:01; HLA-B07:02; HLA-B08:01; HLA-B27:05; HLA-B39:01; HLA-B40:01; HLA-B58:01; HLA-B15:01. The thresholds for the definition of binding were maintained as the tool defaults, only binding results classified as “strong binding” were considered.

### 4.5. HLA Prevalence in the Brazilian Population

The Allele Frequencies Database [87] was used as the source of this information for the Brazilian population. To obtain cohesive and yet representative nationwide results, the bone marrow registry (REDOME) data was selected. Since this data is separated by Brazilian state, we calculated the proportional values for a national level estimation.

### 4.6. Statistical Analysis

Statistical analysis was performed by using Epi Info™ version 7.2.4.0 (https://www.cdc.gov/epiinfo/index.html, accessed on 1 May 2021), relying on Mantel-Haenszel test, at significance level of 0.05, to evaluate statistical correlations. SPSS^®^ Statistics version 26.0 (IBM^®^, Armonk, NY, USA) was also utilized for performing mean comparison with t-test, at a 0.05 significance level.

## Figures and Tables

**Table 1:** Characterization of the study population.

	HIV-1^+^ Individuals	Female	Male
All ages	20,226 (100.00%)	8962 (44.3%)	11,263 (55.7%)
<1 yrs old	64 (0.32%)	30 (0.15%)	34 (0.17%)
2–9 yrs old	364 (1.80%)	200 (0.99%)	164 (0.81%)
10–17 yrs old	840 (4.15%)	426 (2.11%)	414 (2.05%)
18–30 yrs old	2443 (12.08%)	1201 (5.94%)	1242 (6.14%)
30–49 yrs old	12,426 (61.44%)	5390 (26.65%)	7036 (34.79%)
50–79 yrs old	4074 (20.14%)	1709 (8.45%)	2364 (11.69%)
>80 yrs old	15 (0.07%)	6 (0.03%)	9 (0.04%)
Age (av. yrs ± std)	39.55 ± 12.71	38.67 ± 13.15	40.25 ± 12.31
Treatment (av. yrs ± std)	2.98 ± 2.96	3.06 ± 2.96	2.92 ± 2.95
**Birth Federative Unit**	**HIV-1^+^ individuals**	**Treatment Scheme**	**HIV-1^+^ individuals**
São Paulo	4445 (21.98%)	3TC,EFV,TDF	4324 (21.38%)
Rio Grande do Sul	2419 (11.96%)	3TC,AZT,EFV	4003 (19.79%)
Minas Gerais	1878 (9.29%)	3TC,AZT,LPV	2258 (11.16%)
Rio de Janeiro	1861 (9.20%)	3TC,LPV,TDF	1666 (8.24%)
Paraná	1606 (7.94%)	3TC,ATV,RTV,TDF	1355 (6.70%)
Others	8017 (39.64%)	Others	6620 (32.73%)

av. average; std, standard deviation; yrs, years; HIV-1^+^, HIV-1 infected.

**Table 2:** Yearly distribution of the most prevalent surveillance drug-resistance mutations in HIV-1 RT and PR.

	2008	2009	2010	2011	2012	2013	2014	2015	2016	2017 **
M184V	276 (76.88)	416 (73.37)	375 (75.45)	1076 (75.3)	1781 (72.46)	2082 (71.94)	2004 (69.58)	1874 (68.29)	2684 * (53.21)	697 (51.48)
K103N	149 (41.5)	252 (44.44)	217 (43.66)	638 (44.65)	1083 (44.06)	1215 (41.98)	1180 (40.97)	1293 * (47.12)	2145 * (42.53)	566 (41.8)
M41L	125 (34.82)	182 (32.1)	155 (31.19)	383 (26.8)	561 * (22.28)	585 * (20.21)	425 * (14.76)	374 (13.63)	545 * (10.8)	145 (10.71)
D67N	114 (31.75)	163 (28.75)	149 (29.98)	337 * (23.58)	504 * (20.5)	487 * (16.83)	435 (15.1)	398 (14.5)	526 * (10.43)	140 (10.34)
T215Y	109 (30.36)	144 (25.4)	141 (28.37)	299 * (20.92)	403 * (16.4)	434 (15)	323 * (11.22)	277 (10.09)	336 * (6.66)	72 (5.32)
K70R	96 (26.74)	133 (23.46)	131 (26.36)	308 * (21.55)	451 * (18.35)	459 * (15.86)	368 * (12.78)	344 (12.54)	460 * (9.12)	116 (8.57)
V82A	82 (22.84)	111 (19.58)	91 (18.31)	206 * (14.42)	335 (13.63)	313 * (10.82)	251 * (8.72)	242 (8.82)	303 * (6.01)	87 (6.43)
M46I	81 (22.56)	122 (21.52)	89 (17.91)	207 (14.49)	302 (12.29)	302 * (10.44)	222 * (7.71)	207 (7.54)	324 (6.42)	82 (6.06)
L210W	75 (20.89)	109 (19.22)	96 (19.32)	204 * (14.28)	294 * (11.96)	301 (10.4)	199 * (6.91)	172 (6.27)	249 * (4.94)	66 (4.87)
I54V	73 (20.33)	115 (20.28)	79 (15.9)	191 (13.37)	269 * (10.94)	273 (9.43)	224 * (7.78)	196 (7.14)	241 * (4.78)	58 (4.28)
G190A	46 (12.81)	80 (14.11)	47 * (9.46)	146 (10.22)	239 (9.72)	266 (9.19)	271 (9.41)	259 (9.44)	392 * (7.77)	102 (7.53)
P225H	36 (10.03)	56 (9.88)	62 (12.47)	179 (12.53)	320 (13.02)	403 (13.93)	370 (12.85)	390 (14.21)	586 * (11.62)	158 (11.67)
K65R	8 (2.23)	21 (3.7)	23 (4.63)	74 (5.18)	161 (6.55)	194 (6.7)	246 * (8.74)	374 * (13.63)	589 * (11.68)	164 (12.11)

Percentage number under parentheses. * A statistical difference was found with data from the previous year (*p* < 0.05). ** Data for 2017 was only available Jan–Apr.

**Table 3:** K65R mutant strong binding affinity to most frequent HLAs.

	WT SB Binding	K65R SB Binding	Allele Freq. in BR Pop.
HLA-A01	no	no	9.21
HLA-A02	no	no	25.94
HLA-A03	yes	yes	9.26
HLA-A24	no	no	10.00
HLA-A26	no	no	3.35
HLA-B07	no	no	6.92
HLA-B08	no	no	5.12
HLA-B15	no	no	9.08
HLA-B27	no	yes	2.23
HLA-B39	no	no	3.46
HLA-B40	no	no	4.70
HLA-B58	yes	yes	2.65

## Data Availability

The HIV-1 sequences selected for this study were made available in GenBank (accession numbers will be included here).

## Supplementary Materials

The following are available online at https://www.mdpi.com/article/10.3390/ijms22105304/s1, Figure S1: Presence of SDMR among subtypes, Figure S2: Phylogenetic representation of a subset of HIV-1 subtype C sequences isolated in Brazil with the K65R mutation and closely related sequences from databases, Figure S3: Phylogenetic representation of a subset of HIV-1 subtype B sequences isolated in Brazil with the K65R mutation and closely related sequences from databases. Table S1: Phylogeny inferred HIV-1 transmission clusters with more than one sequences harboring the K65R mutation, Table S2: Top SDRM on different Brazilian Regions through the years.
