# Supplementary material for: Nationwide Study of Drug Resistance Mutations in HIV-1 Infected Individuals under Antiretroviral Therapy in Brazil
Source: Int J Mol Sci. 2021 May 18;22(10):5304. doi: 10.3390/ijms22105304 (PMC8157590; doi:10.3390/ijms22105304)
Supplement: Supplementary file 1 [file ijms-22-05304-s001.zip › SantosPereiraetal_supdata_V2.pdf]

Nationwide study of drug resistance mutations in HIV-1 infected individuals under antiretroviral therapy in Brazil

Ana Santos-Pereira[1,2], Vera Triunfante[1,2], Pedro M.M. Araújo[1,2], Joana Martins[1,2], Helena Soares[4,5], Eva Poveda[6], Bernardino Souto[1,2,3] and Nuno S. Osório[1,2*]

[1] Life and Health Sciences Research Institute (ICVS), School of Medicine, University of Minho, Braga, Portugal
[2] ICVS/3B's - PT Government Associate Laboratory, Braga, Guimarães, Portugal
[3] Department of Medicine, Federal University of São Carlos, São Paulo, Brazil
[4] Human Immunobiology and Pathogenesis Laboratory, Lisbon, Portugal
[5] CEDOC-Chronic Diseases Research Center, NOVA Medical School | Faculdade de Ciências Médicas, NOVA University of Lisbon, Lisbon, Portugal
[6] Group of Virology and Pathogenesis, Galicia Sur Health Research Institute (IIS Galicia Sur)-Complexo Hospitalario Universitario de Vigo, SERGAS-UVigo, Vigo, Spain
* Correspondence: nosorio@med.uminho.pt; Tel: 351-253-604904

Supplementary Data

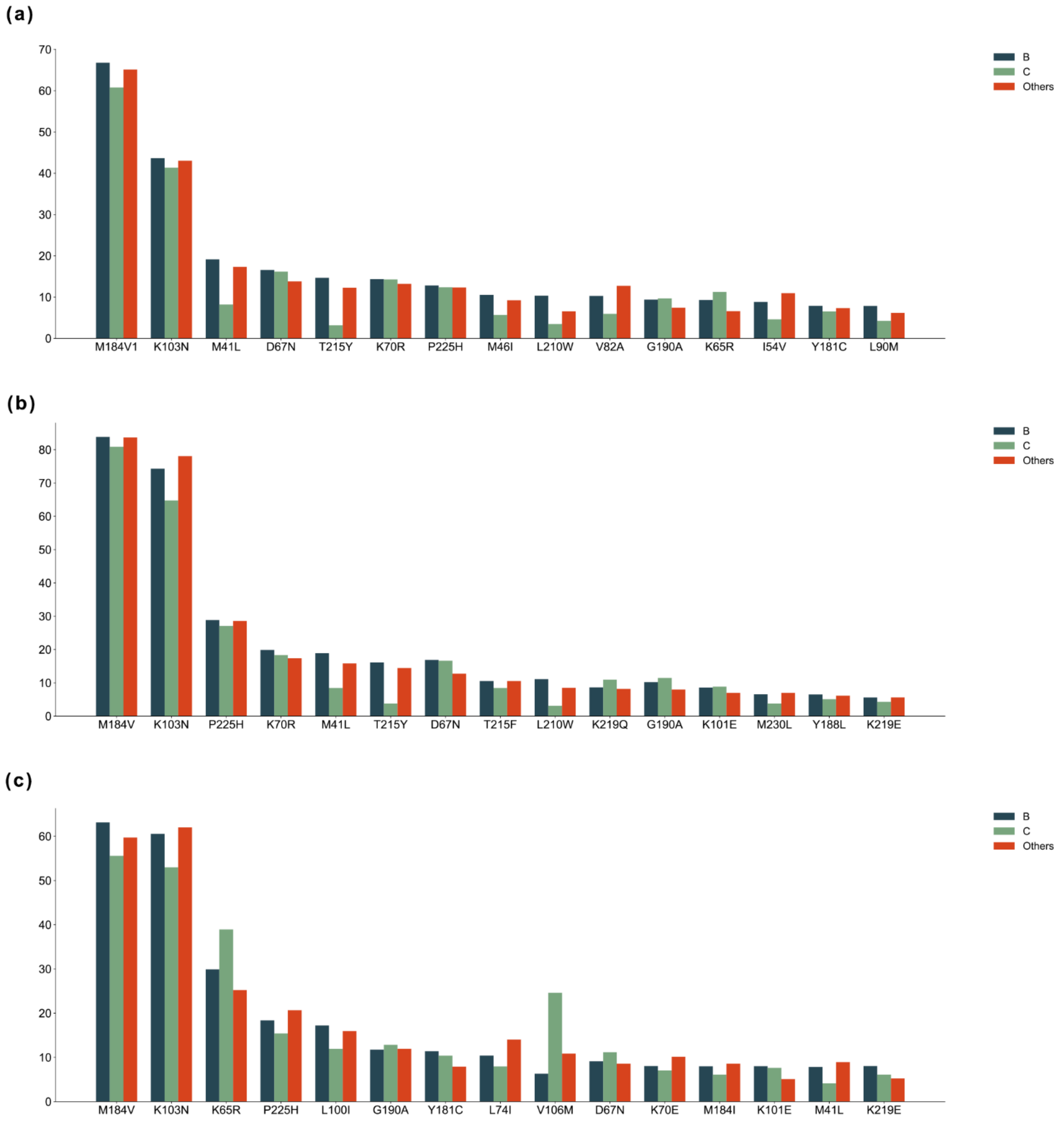

Figure S1. Presence of SDMR among subtypes. Top 15 most prevalent SMDR among different HIV-1 subtypes was evaluated in all the individuals of the cohort (a), in patients receiving a treatment scheme including AZT (b) or TDF (c).

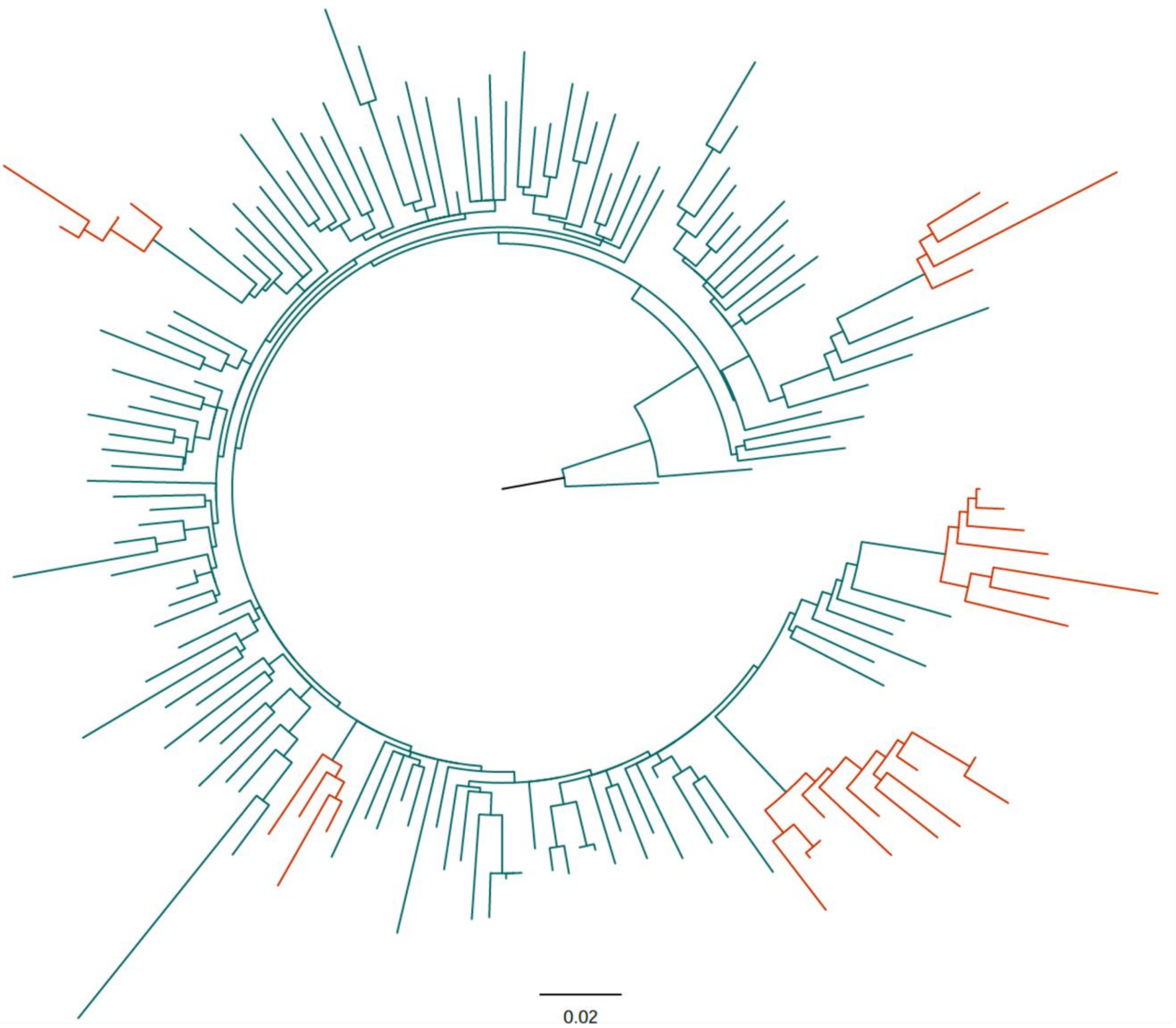

Figure S2. Phylogenetic representation of a subset of HIV-1 subtype C sequences isolated in Brazil with the K65R mutation and closely related sequences from databases. Circular cladogram representation of the Maximum likelihood tree. Branch colored in red indicate inferred transmission clusters. The tree was rooted considering references of subtype A1. To improve tree visualization a separate analysis excluding the outgroup sequences is shown.

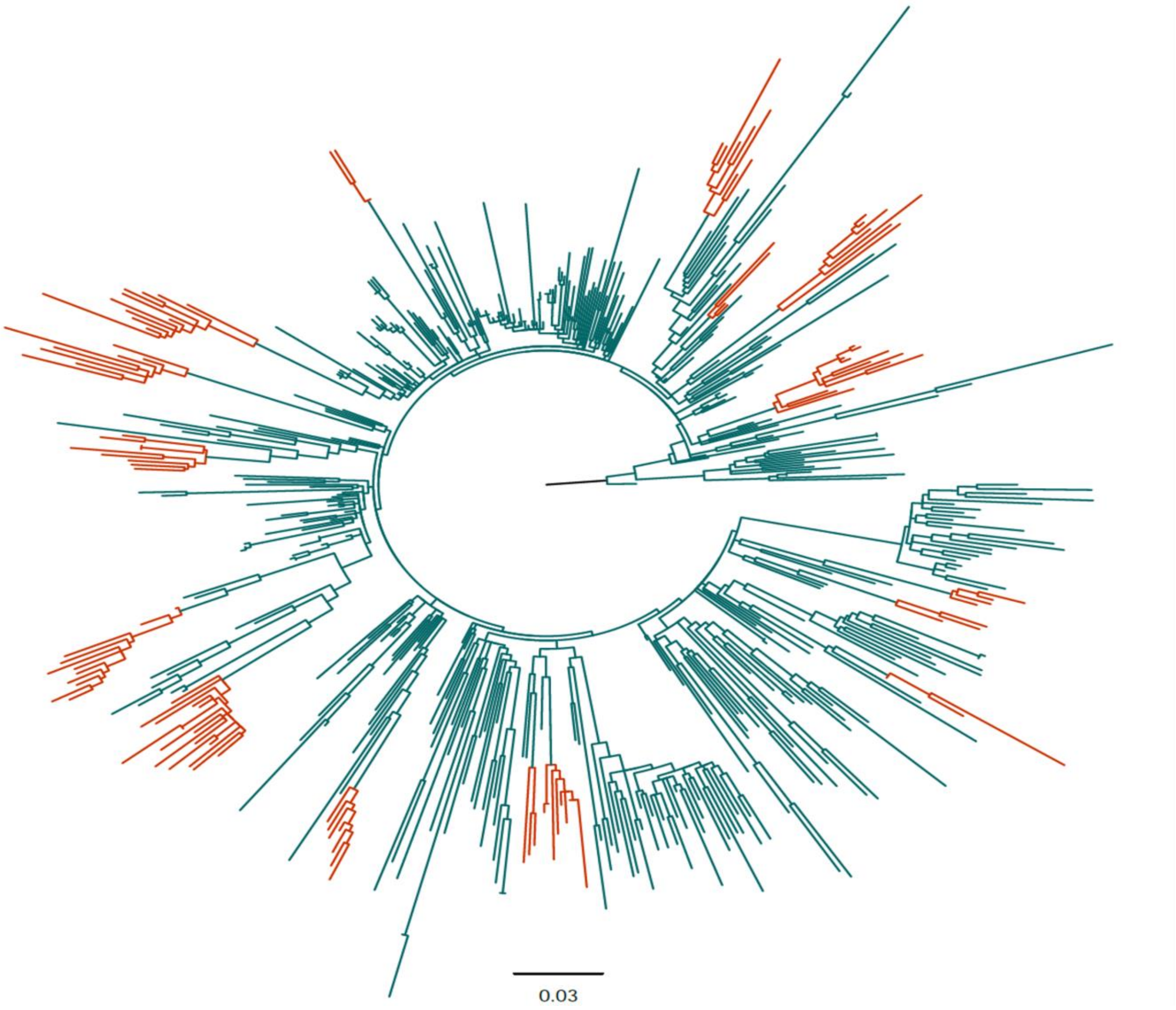

Figure S3. Phylogenetic representation of a subset of HIV-1 subtype B sequences isolated in Brazil with the K65R mutation and closely related sequences from databases. Circular cladogram representation of the Maximum likelihood tree. Branch colored in red indicate inferred transmission clusters. The tree was rooted considering references of subtype A1. To improve tree visualization a separate analysis excluding the outgroup sequences is shown.

Table S1. Phylogeny inferred HIV-1 transmission clusters with more than one sequences harboring the K65R mutation.

Sequence	Origin	Cluster	Sample Year	Country	Federative Unit*	Transmission route	Gender	Presence of K65R mutation
18SC110074	This study	B1	2011	Brazil	SC	Homosexual	Male	No
21PR130051	This study	B1	2013	Brazil	PR	Bisexual	Male	No
21PR140146	This study	B1	2014	Brazil	PR		Male	Yes
21PR140647	This study	B1	2014	Brazil	PR		Male	Yes
26PR150111	This study	B1	2015	Brazil	PR	Homosexual	Male	No
29PR1604451	This study	B1	2016	Brazil	PR	Homosexual	Male	No
29PR1610702	This study	B1	2016	Brazil	PR		Male	No
24PI150732	This study	B2	2015	Brazil	PI		Male	Yes
29PI1700952	This study	B2	2017	Brazil	PI		Male	Yes
KF782694	Public Databases	B2	2012	Brazil	MA	Homosexual	Male	No
29SP1606275	This study	B3	2016	Brazil	SP	Heterosexual, Drugs	Male	No
29SP1608861	This study	B3	2016	Brazil	SP	Homosexual	Male	Yes
29SP1610014	This study	B3	2016	Brazil	SP	Heterosexual	Female	Yes
11SP151909	This study	B4	2015	Brazil	SP		Male	Yes
29ES1700443	This study	B4	2017	Brazil	ES	Bisexual	Male	No
29MG1601542	This study	B4	2016	Brazil	MG		Male	Yes
29DF1701982	This study	B5	2017	Brazil	DF	Homosexual	Male	Yes
29PA1605620	This study	B5	2016	Brazil	PA		Male	Yes
29PA1608064	This study	B5	2016	Brazil	PA		Male	Yes
29PA1608071	This study	B5	2016	Brazil	PA	Heterosexual	Male	Yes
29PA1702877	This study	B5	2017	Brazil	PA		Female	Yes
KX887674	Public Databases	B5	2015	Brazil	PA			No
KX887675	Public Databases	B5	2015	Brazil	PA			No
11SP140792	This study	B6	2014	Brazil	SP		Female	Yes
29RJ1608399	This study	B6	2016	Brazil	RJ		Male	No
29SP1609861	This study	B6	2016	Brazil	SP	Heterosexual	Male	Yes
29SP1610943	This study	B6	2016	Brazil	SP	Homosexual	Male	Yes
29SP1701160	This study	B6	2017	Brazil	AL		Male	No
JN100838	Public Databases	B6	2003	United Kingdom		Homosexual	Male	No
JN195956	Public Databases	B6	2008	Brazil	SP			No
KT427797	Public Databases	B6	2010	Brazil	SP			No
04DF150339	This study	B7	2015	Brazil	PB		Male	No
29RS1600171	This study	B7	2016	Brazil	RS		Male	Yes
EU248364	Public Databases	B7	2003	Belgium				No
FJ228082	Public Databases	B7	2006	Italy				No
FJ228130	Public Databases	B7	2004	Italy				No
GQ399355	Public Databases	B7	2005	Portugal				No

GU969527	Public Databases	B7	2008	Italy			Male	No
JF487842	Public Databases	B7	2008	Brazil	RS			No
JX299665	Public Databases	B7	2007	Germany				No
MN972080	Public Databases	B7	2015	Brazil	MA			Yes
MT570426	Public Databases	B7	2015	United Kingdom				No
MT787777	Public Databases	B7	2008	Italy				No
MT787784	Public Databases	B7	2007	Italy				No
09CE140280	This study	B8	2014	Brazil	CE	Heterosexual	Female	Yes
21PR120215	This study	B8	2012	Brazil	PR		Male	Yes
EU340735	Public Databases	B8	2006	Brazil	PR			No
FJ591477	Public Databases	B8	2006	Brazil	PR			No
11PB150082	This study	B9	2015	Brazil	PB	Homosexual	Male	Yes
29MG1602837	This study	B9	2016	Brazil	MG		Male	No
29PE1601370	This study	B9	2016	Brazil	PE		Male	No
29PE1602220	This study	B9	2016	Brazil	PE	Homosexual	Male	No
29PE1605080	This study	B9	2016	Brazil	PE		Male	Yes
29PE1609931	This study	B9	2016	Brazil	PE	Heterosexual	Female	Yes
29PE1610633	This study	B9	2016	Brazil	PE		Male	Yes
29PE1610635	This study	B9	2016	Brazil	PE	Homosexual	Male	No
29PE1610642	This study	B9	2016	Brazil	PE	Bisexual	Male	No
29RJ1606703	This study	B9	2016	Brazil	RJ		Male	No
KJ849784	Public Databases	B9	2010	Brazil	PE			No
06MG140069	This study	B10	2014	Brazil	MG		Male	No
09CE140292	This study	B10	2014	Brazil	CE	Homosexual	Male	Yes
11SP140667	This study	B10	2014	Brazil	SP		Male	Yes
11SP140670	This study	B10	2014	Brazil	SP	Homosexual	Male	No
29CE1605374	This study	B10	2016	Brazil	CE		Male	Yes
29DF1605721	This study	B10	2016	Brazil	DF	Heterosexual	Male	No
29RJ1606078	This study	B10	2016	Brazil	RJ		Male	Yes
29SP1610234	This study	B10	2016	Brazil	SP	Homosexual	Male	No
29SP1702293	This study	B10	2017	Brazil	CE	Homosexual	Male	No
KP115562	Public Databases	B10	2014	Brazil	SP			No
KX661656	Public Databases	B10	2011	United Kingdom				No
KX888117	Public Databases	B10	2015	Brazil	GO			No
KX888368	Public Databases	B10	2015	Brazil	MG			No
KX888759	Public Databases	B10	2015	Brazil	SP			No
KX888774	Public Databases	B10	2015	Brazil	RJ			No
KX889064	Public Databases	B10	2015	Brazil	SC			No
06BA150083	This study	B11	2014	Brazil	BA		Male	No
06BA150203	This study	B11	2015	Brazil	SE	Homosexual	Male	Yes
06BA150391	This study	B11	2015	Brazil	BA		Male	No

29BA1603668	This study	B11	2016	Brazil	BA	Heterosexual	Female	No
29BA1605576	This study	B11	2016	Brazil	BA	Bisexual	Male	Yes
29BA1605660	This study	B11	2016	Brazil	BA		Male	No
29BA1701551	This study	B11	2017	Brazil	BA		Male	No
29SP1702999	This study	B11	2017	Brazil	BA	Heterosexual	Male	Yes
22MS140168	This study	B12	2014	Brazil	MS		Male	Yes
29MS1604163	This study	B12	2016	Brazil	MS		Male	Yes
29PR1610894	This study	B12	2016	Brazil	PR		Male	Yes
JF342308	Public Databases	B12	2008	Brazil	MS			No
JF342321	Public Databases	B12	2010	Brazil	MS			No
JN196004	Public Databases	B12	2010	Brazil	SP			No
KJ849805	Public Databases	B12	2010	Brazil	SP			No
MN528456	Public Databases	B12	2017	Brazil	AM			No
MN528463	Public Databases	B12	2017	Brazil	AM			No
10SP151332	This study	B13	2015	Brazil	SP		Male	Yes
29SP1603165	This study	B13	2016	Brazil	SP	Bisexual	Male	Yes
29SP1700487	This study	B13	2017	Brazil	SP		Male	No
06MG130344	This study	B14	2013	Brazil	RJ		Male	No
09RJ130607	This study	B14	2013	Brazil	RJ		Male	Yes
11SP138750	This study	B14	2013	Brazil	SP		Male	No
11SP151590	This study	B14	2015	Brazil	SP	Homosexual	Male	No
29MG1611880	This study	B14	2016	Brazil	MG		Male	Yes
29RJ1610193	This study	B14	2016	Brazil	RJ	Homosexual	Male	No
29SP1601805	This study	B14	2016	Brazil	SP		Male	No
KX662590	Public Databases	B14	2010	United Kingdom				No
LC162092	Public Databases	B14	2013	Japan				No
LC162094	Public Databases	B14	2013	Japan				No
MN163692	Public Databases	B14	2014	Croatia				No
04DF130122	This study	B15	2013	Brazil	DF		Male	Yes
29DF1600435	This study	B15	2016	Brazil	DF		Male	No
29DF1701062	This study	B15	2017	Brazil	DF	Homosexual	Male	Yes
29RS1703532	This study	B15	2017	Brazil	RS		Male	No
KX888083	Public Databases	B15	2015	Brazil	DF			No
KX888084	Public Databases	B15	2015	Brazil	DF			No
KX888087	Public Databases	B15	2015	Brazil	DF			No
LC526723	Public Databases	B15	2017	Japan				No
MT570579	Public Databases	B15	2016	United Kingdom				No
12SP091875	This study	B16	2009	Brazil	SP		Male	Yes
23SP110075	This study	B16	2011	Brazil	SP	Homosexual	Male	Yes
HM534048	Public Databases	B16	2009	Brazil	SP			No
HM534096	Public Databases	B16	2009	Brazil	SP			No

11SP139055	This study	C1	2013	Brazil	SP		Male	Yes
21PR110049	This study	C1	2011	Brazil	PR	Homosexual	Male	No
21PR130023	This study	C1	2013	Brazil	PR	Bisexual	Male	No
29PR150058	This study	C1	2015	Brazil	PR		Male	Yes
15SP131030	This study	C2	2013	Brazil	SP	Bisexual	Male	Yes
29TO1610854	This study	C2	2016	Brazil	TO	Homosexual	Male	Yes
FJ784207	Public Databases	C2	2003	Brazil	GO		Female	No
KT744431	Public Databases	C2	2009					No
29TO1611348	This study	C2	2016	Brazil	TO		Male	No
29MG1609380	This study	C2	2016	Brazil	MG	Heterosexual	Male	No
JN114145	Public Databases	C2	2009	Brazil	GO		Female	No
07SC140751	This study	C3	2014	Brazil	SC	Bisexual	Male	No
21PR130350	This study	C3	2013	Brazil	PR	Heterosexual	Male	Yes
29SP1701779	This study	C3	2017	Brazil	SP		Male	Yes
EU340722	Public Databases	C3	2005	Brazil	PR			No
29SP150078	This study	C4	2015	Brazil	SP		Male	No
29SP1701889	This study	C4	2017	Brazil	SP		Male	Yes
29SP1702463	This study	C4	2017	Brazil	SP	Heterosexual	Male	Yes
KX888620	Public Databases	C4	2015	Brazil	SP			No
26PR150119	This study	C5	2015	Brazil	PR		Male	Yes
29MS1701288	This study	C5	2017	Brazil	MS	Heterosexual	Male	No
29SP1611091	This study	C5	2016	Brazil	SP	Bisexual	Male	No
29SP1700076	This study	C5	2017	Brazil	SP	Heterosexual	Male	Yes
29SP1700481	This study	C5	2017	Brazil	SP		Male	No
29SP1702372	This study	C5	2017	Brazil	SP		Female	Yes
JN196005	Public Databases	C5	2010	Brazil	SP			No
JN196007	Public Databases	C5	2010	Brazil	SP			No
KT427806	Public Databases	C5	2010	Brazil	SP			No
KY640158	Public Databases	C5	2015	Brazil	SP			Yes
MT787796	Public Databases	C5	2008	Italy				No

***** SC – Santa Catarina, PR – Paraná, PI – Piauí, MA – Maranhão, SP – São Paulo, ES – Espírito Santo, MG – Minas Gerais, DF – Distrito Federal, PA – Pará, RJ – Rio de Janeiro, AL – Alagoas, PB – Paraíba, RS – Rio Grande do Sul, CE – Ceará, PE – Pernambuco, GO – Goiás, BA – Bahia, SE – Sergipe, MS – Mato Grosso do Sul, AM – Amazonas, TO – Tocantins.
